# Utilizing Infantile Spasm Seizure Activity as a Baseline Vital in the Setting of Acute Pseudomonas aeruginosa Pneumonia

**DOI:** 10.7759/cureus.46269

**Published:** 2023-09-30

**Authors:** Landon R Thompson, Richard Virgilio, David L Flowers

**Affiliations:** 1 Medicine, Edward Via College of Osteopathic Medicine, Auburn, USA; 2 Clinical Affairs, Edward Via College of Osteopathic Medicine, Auburn, USA; 3 Pediatric Medicine, Piedmont Columbus Regional Hospital Midtown Campus, Georgia, USA

**Keywords:** infantile spasms, pseudomonas pneumonia, seizures, hpa axis, acth

## Abstract

The objective of this case report is to describe and document a decrease in seizure activity in a 16-year-old female with a past medical history of Aicardi syndrome (AS) and infantile spasms (IS) while being treated for acute* Pseudomonas aeruginosa* pneumonia with pleural effusion. This patient presented to the pediatric emergency department with a chief complaint of fever, tachycardia, increased nasal secretions, and oxygen requirement at home. She was admitted to the general pediatric medical floor for treatment of an adenovirus infection due to her having a complex medical history and her being medically unstable. On hospital admission day 1, she developed post-viral *P. aeruginosa* pneumonia. She subsequently had three days of complete clinical seizure cessation without changing her anti-epileptic medications. It was not until the symptomatology related to her pneumonia improved that her seizure activity returned to its baseline frequency. The treating team discovered that the decrease in her frequency of seizure activity related to periods of increased physiologic stress was not new. Her mother reported that she has used the relationship between her daughter’s seizures and any acute illness to gauge how her daughter was “feeling” medically. Three weeks prior to this hospital admission, her mother reported that her daughter’s seizures ceased for two days during a period in which it was determined that the patient was having renal colic and passed a renal stone. This phenomenon, the decrease in the frequency of seizure activity related to periods of increased physiologic stress, could help primary caretakers assess when significant, new comorbid conditions are present and could aid in the primary assessment of physical health in a particular patient population who are unable to verbalize their current medical status. Utilizing seizure activity as an at-home vital sign could help caretakers recognize when their patient is under an elevated physiologic stress condition. Recognizing the relationship between seizure frequency and acute illness could also help diagnostically, as ISs are difficult to both diagnose and manage. Also, future research on this possible association could explore more understanding of IS and pathophysiology of such phenomenon.

## Introduction

Aicardi syndrome (AS) is an extremely rare and devastating neurodevelopmental disorder characterized by callosal agenesis, infantile spasms, and chorioretinal lacunae [1]. The estimated incidence rate of AS in the United States is one in 105,000-167,000 [2]. The mean survival time is over 30 years and varies based on severity [3]. The mortality rate of AS is highest during the first years of life, but the survival rate improves as pediatric patients’ age increases [3]. At 25 years of age, the probability of surviving another five years is estimated to be 85% [3]. The most common causes of death in these patients are acute respiratory failure and infection [4]. The gene responsible for AS is unknown [1]. While most cases are de novo, mostly females and Klinefelter (XXY) males are affected, suggesting involvement of the X chromosome in a dominant pattern [5]. This mutation is presumed lethal in utero to XY males, as no affected XY males have been identified [1].

Infantile spasms (IS) are a type of epileptic seizure characterized by clusters of myoclonic spasms starting with a slight head flexion [6]. IS leads to regression of neurological development, and uncontrolled seizure activity leads to devastating long-term neurocognitive effects [7]. It is theorized that the hallmark hypsarrhythmia on EEG is responsible for the intellectual disability following these seizures [7]. The etiology of IS is difficult to ascertain and can be classified as symptomatic, cryptogenic, or idiopathic [8]. Symptomatic IS has a known structural, metabolic, or genetic etiology and occurs in children with developmental delays prior to the onset of spasms [8]. The pharmacologic therapy depends on the IS etiology if identified, calling for a thorough and detailed diagnostic approach [8]. This case report focuses on a patient with symptomatic IS due to AS.

Currently, adrenocorticotropic hormone (ACTH) and vigabatrin are first-line therapies for infantile spasms [5]. Vigabatrin therapy shows a higher efficacy in treating IS secondary to tuberous sclerosis [9]. There is no standard dosing recommendation regarding ACTH [10]. The evidence for dosing ACTH is varied in the literature, especially considering the treatment is dependent on etiology [11]. Different studies utilize different parameters [11]. For example, high-dose ACTH in one study is sometimes lower than the low-dose ACTH used in another study [11]. Without universally defined boundaries for these terms across studies, it is difficult to build conclusive evidence to support a standard therapeutic window [11]. Prescribing a high dose, supraphysiologic regimen of ACTH results in more severe and frequent adverse effects such as arrhythmias, elevated blood pressure, and electrolyte abnormalities [12]. Moderate evidence suggests that low-dose ACTH is just as efficacious as high-dose ACTH [13]. The goal of treatment is complete amelioration of seizures and resolution of hypsarrhythmia on EEG while attempting to minimize adverse effects [7].

The hypothalamus controls the regulation and function of the endocrine system [14]. It is responsible for the synthesis and secretion of multiple hormones, specifically cortisol-secreting hormone (CRH) [14]. CRH causes an increase in the secretion of ACTH via the anterior pituitary [15]. This ACTH proceeds to the adrenal glands, where it stimulates the zona fasciculata layer of the cortex to secrete cortisol [16]. Cortisol is a glucocorticoid hormone described as the stress hormone [17]. The serum concentration of cortisol increases in times of acute, perceived stress [17]. The end effects of cortisol include increased carbohydrate synthesis from protein, increased blood pressure, insulin resistance, gluconeogenesis, inflammatory and immune responses, and bone formation [18]. When serum cortisol increases, negative feedback to the hypothalamus and the anterior pituitary decreases the amount of CRH and ACTH, decreasing cortisol secretion [19]. The effects of cortisol are rapidly achieved upon secretion, exerting a quick onset of action via intracellular receptor binding [20].

This case report follows the hospital course and treatment of a patient with post-viral *Pseudomonas aeruginosa* pneumonia and a past medical history of IS secondary to AS. During her illness, there was a complete cessation of seizure activity for over four days followed by a complete return to baseline seizure activity toward the end of therapy. The patient's mother reported multiple past incidents of this phenomenon, which she has used to vigilantly gauge the onset of an acute comorbid illness affecting her daughter. In patients who still exhibit infantile spasm seizure activity, this correlation could prove helpful in assessing their daily baseline seizure activity so that deviations can be noted. Deviations from baseline could possibly indicate a change in health due to acute illness or pathology. The transient amelioration and relapse of this patient’s seizures during a time of illness could function to provide valuable insight into the current physical health of the patient, who otherwise cannot verbalize how she feels.

## Case presentation

A 16-year-old female presented to a community hospital pediatric emergency department (ED) accompanied by her mother, who serves as the primary caretaker and provider of medical history, with a primary concern of persistent fever, increased heart rate, increased nasal secretions, and increased oxygen requirement at home from bilevel positive airway pressure (BiPAP) only at night to BiPAP waking and sleeping hours. Her past medical history is significant for AS, IS, and cerebral palsy. She is treated with nightly supplemental oxygen via BiPAP for obstructive sleep apnea (OSA) and is gastronomy tube (G-tube) dependent. At home baseline, she utilizes BiPAP only at night. Her inspiratory pressure is set to 14 cmH_2_O and her expiratory pressure is set to 6 cmH_2_O with no required daily oxygen therapy. Her current medications include baclofen, nebulized budesonide, calcium-vitamin D, cannabidiol, cetirizine, clobazam, ipratropium bromide/albuterol, rufinamide, sodium citrate, topiramate, and valproate. The patient's reported medication allergies are cefdinir, trimethoprim/sulfamethoxazole, levofloxacin, and meropenem. The reaction the patient encounters with these drugs is all the same: increased seizures. The patient was initially evaluated for the above-listed presenting symptoms in an outpatient setting and was treated with amoxicillin/clavulanate for a presumed upper respiratory infection. After completing the prescribed seven-day course of amoxicillin/clavulanate, she had minimal improvement in her respiratory symptoms, per the patient's mother. The patient experienced oxygen desaturation, prompting her mother to bring her to the pediatric ED by ambulance for evaluation. At the time she presented to the ED, she was experiencing baseline seizure activity of six to eight seizures daily, moderate respiratory distress, persistent tachycardia, green nasal secretions, and a low-grade fever of 100.4°F. Her at-home medications were continued as directed. The low-grade fever of 100.4°F persisted in the ED despite treatment with alternating acetaminophen and ibuprofen. A mobile chest radiography, shown below, ordered in the ED showed a left lower lobe infiltrate with mild pleural effusion. A respiratory panel was significant for adenovirus. She was admitted to the general pediatric medical floor for treatment of an adenovirus infection because she was medically unstable, with a complex medical history. Respiratory therapy was also consulted to manage supplemental oxygen administration. 

**Figure 1:**
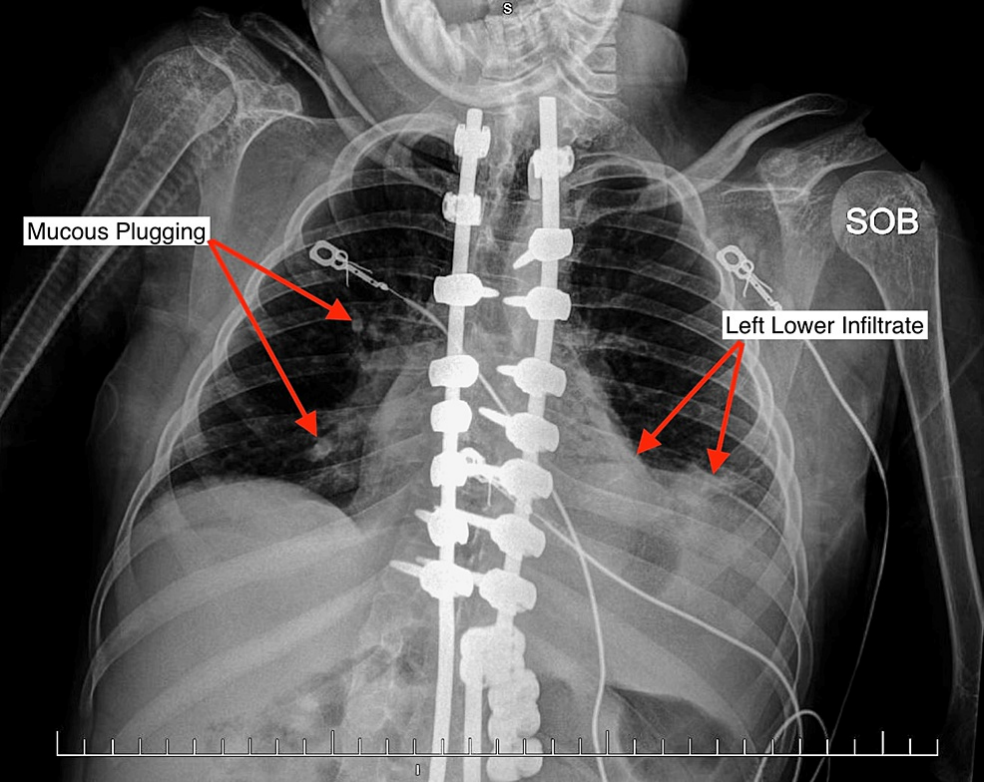
This is the patients first chest radiography, taken in the ED. The findings, labeled on the radiograph, were remarkable for a left lower lobe infiltrate, mild left-sided pleural effusion, and moderate mucous plugging.

On admission day 1, based on the patient's chest x-ray results and her physical exam findings, she was empirically treated for Streptococcus pneumoniae pneumonia with clindamycin, a decision that took into consideration her allergy to cephalosporins and the rare cross-reactivity that can occur with penicillin and her recently finished course of amoxicillin/clavulanate, which resulted in minimal symptom alleviation. Supportive therapy with inhaled ipratropium bromide/salbutamol and regular chest physiotherapy were also started. The patient required 2 liters of oxygen via a nasal cannula during the day and via her BiPAP at night. This is an increased oxygen demand from the baseline of only nightly use. No seizure activity was noted by the inpatient pediatric team at that time. Her physical exam at this time was significant for increased work of breathing, bilaterally decreased lung sounds, wheezing, and increased oxygen requirement. That night, broader coverage was advised by the pediatric team due to minimal clinical improvement on clindamycin. Therefore, vancomycin was started.

By admission day 2, the patient's clinical exam improved slightly with better work of breathing and improved breath sounds. The respiratory culture showed moderate growth of *Pseudomonas aeruginosa*. The pharmacy was consulted to determine the best coverage in the setting of the patient's allergies list. The patient’s antibiotics were switched to aztreonam and gentamicin. Clindamycin and vancomycin were discontinued. Prednisolone was added to her therapy via G-tube. Overnight, the mother reported some loose stools, for which loperamide was prescribed on an as-needed basis.

On the morning of admission day 3, the patient's pediatric pulmonologist, whom she sees regularly, was consulted regarding the best method to cover this patient's pneumonia. Ultimately, it was decided that single coverage for Pseudomonas would suffice. Therefore, the patient was continued on aztreonam with no other antibiotics added. There was no seizure activity noted by the pediatric team, floor nurses, or mother since the patient’s hospital admission. Her respiratory exam showed further improvement compared to earlier admission days. Throughout the night of admission day 3, the patient was able to maintain adequate oxygen saturation with daily and nightly supportive oxygen therapy and plans were made to discharge her to home once she was no longer requiring daytime oxygen therapy. Home Health, a nursing agency that aids in healthcare tasks and continuation of care in the patient's home setting, was also consulted for the continued antibiotic administration post-discharge.

On admission day 4, a midline venous catheter was placed to assist with at-home aztreonam administration. She was also successfully weaned from daily oxygen therapy. The patient’s work of breathing was significantly improved, and breath sounds on the exam were clear and equal. The patient’s mother consented to discharge her with at-home administration of aztreonam and prednisolone. Case management set up delivery of the aztreonam to the patient’s home. It was on this night that the patient exhibited her first seizure since being admitted to the pediatric inpatient unit four days prior. At this time her mother even mentioned she believed that this meant her daughter was feeling much better. The patient had one more seizure during this night.

On the morning of admission day 5, the patient had two more seizures and one minor desaturation which was promptly treated with her BiPAP. The patient was deemed medically stable for discharge by the pediatric team due to her return to baseline oxygen requirement and much improved physical exam findings.

## Discussion

AS is rare; however, other aspects of this case raised scientific interest. Per this patient's mother, the patient's baseline seizure activity is six to eight seizures daily. How did the patient achieve significant resolution of seizure activity without changing any of her antiepileptic therapy regimens? Because of her immense and diverse past medical history coupled with an exhaustive medication list, it is difficult to prove a reason for this phenomenon. It could be hypothesized that increased endogenous cortisol release, as would be expected in acute illness, infection, or a state of increased physiologic stress related to her *P. aeruginosa* pneumonia, could be the possible cause of this phenomenon [17].

The clinical application of this case report could possibly be utilized by caretakers and clinicians. Caretakers could measure and track seizure activity in the same fashion they measure and track heart rate, respiratory rate, and core body temperature. This may be another data point to ensure proper and timely management of acute illness and increased physiologic stress in a very specific patient population that cannot otherwise verbalize changes in their medical condition. For clinicians, this finding, especially if commonly found and documented in other patients with AS, could help diagnose IS just as methacholine challenge is used to help diagnose asthma or secretin stimulation test is used for diagnosis of gastrinoma. Seizure-like activity could be compared to baseline in times of acute illness to gauge the stress the patient is feeling. As far as treatment is concerned, lower-dose ACTH may be effective in individuals who exhibit this phenomenon. The lowest effective dose of ACTH should be pursued to limit the common and strong adverse effects of high-dose ACTH.

While this phenomenon is reoccurring in this single patient, as per the mother's anecdotal history, there are limitations to this report. Her pneumonia was the primary focus of her visit, thus there was no EEG completed to assess clinical seizure activity. There were also no serum cortisol levels drawn during her hospitalization as the treating team's thoughts on this case did not begin to formulate until the tail end of her visit. There is also the argument that she was given numerous antibiotics that could have contributed to the decrease in clinical seizure activity. Another important consideration is the lack of significant reporting of this phenomenon in the current literature.

## Conclusions

A 16-year-old, nonverbal, Caucasian female on antiepileptics has a history of six to eight IS per day secondary to AS. During times of illness or stress, she repeatedly reduces her clinical seizure activity or even ceases her clinical seizure activity while no changes are made to her antiepilepsy regimen. This phenomenon could aid in the primary assessment of physical health in a small patient population that cannot verbalize their well-being. Also, future research on this possible association could explore more understanding of IS and the pathophysiology of such phenomenon.
